# Protein metabolic changes and nucleolus organizer regions activity in the lymphocytes of neonatal calves during the development of respiratory diseases

**DOI:** 10.14202/vetworld.2019.1657-1667

**Published:** 2019-10-28

**Authors:** Elena Kalaeva, Vladislav Kalaev, Ksenia Efimova, Anton Chernitskiy, Vladimir Safonov

**Affiliations:** 1 Department of Biophysics and Biotechnology, Faculty of Medicine and Biology, Voronezh State University, Voronezh, Russia; 2 Department of Genetics, Cytology and Bioengineering, Faculty of Medicine and Biology, Voronezh State University, Voronezh, Russia; 3 Laboratory of Diseases of the Reproductive Organs, Breast and Young Farm Animals, All-Russian Veterinary Research Institute of Pathology, Pharmacology and Therapy, Voronezh, Russia; 4 Laboratory of Environmental Biogeochemistry, Vernadsky Institute of Geochemistry and Analytical Chemistry of the Russian Academy of Sciences, Moscow, Russia

**Keywords:** bronchopneumonia, calf, creatinine, nucleolus, serum immunoglobulin, serum total protein, urea

## Abstract

**Background and Aim:**

Calfhood disease is an important problem in dairy farming that could cause significant effects on heifer survival and productivity and has economic and welfare effects. Total protein concentration in the blood serum could be one of the predictors of bovine respiratory disease (BRD) in newborn calves. The number of active nucleolus organizers could be used to assess the viability of the protein synthesis system in cells and tissues. We aimed for a comparative assessment of the dynamics of the main indicators of protein metabolism and nucleolus organizer regions (NORs) activity in the lymphocytes of healthy calves (Group I) and calves with BRD (Group II) during the 1^st^ month after birth

**Materials and Methods:**

This study included 30 calves of the red-motley Holstein breed. Venous blood samples were taken from all calves on the 1^st^, 7^th^, 14^th^, and 28^th^ days after birth. Quantitative analysis of total protein (Serum total protein [STP]), immune globulin (Serum immune globulin [SIg]), urea, and creatinine in serum and transcriptionally active chromosome NORs in the interphase nuclei of lymphocytes was conducted using receiver operating characteristic analysis and factor analysis.

**Results:**

In Group I, the STP levels decreased during the 1^st^ month of life, and in Group II, the STP levels were variable. The STP levels in both groups remained within the reference intervals. During the first 2 weeks after birth, the calves’ SIg fluctuated within the statistical error limits and did not significantly differ between the groups. On the 28^th^ day, SIg increased in both the groups (by 42.8% for Group I and 33.7% for Group II). The creatinine concentration showed a decrease but did not go beyond the range of reference values. Urea concentration in Group I markedly decreased and remained below the reference values; it did not change in Group II over the entire observation period. The number of NORs in 1-day-old calves did not significantly differ between the groups and amounted to 2.43 in Group I and 2.59 in Group II. A significant increase in the number of active NORs was found in calves in both groups at the ages of 14 and 28 days. Early BRD predictors (at 1-14 days) could not be identified among the studied indicators. The urea and creatinine concentrations and the NOR activity on day 28 after birth could be late BRD predictors. Protein metabolism in the newborn calves’ organisms is regulated by three types of factors: Maintenance of a constant protein concentration in the plasma, protein decomposition, and *de novo* synthesis.

**Conclusion:**

There were no observed significant differences in the protein metabolism values and dynamics of indicators between healthy calves and calves with developed BRD. Alterations in the studied characteristics are the result, but not the cause of BRD. The increase in active NORs under BRD could be a favorable forecasting indicator. Protection against foreign protein and genetic material is a more important task for the organism than ensuring growth processes during the neonatal period.

## Introduction

Calfhood disease is an important problem in dairy farming that could cause significant effects on heifer survival and productivity and that has economic and welfare effects. Neonatal calf diarrhea (NCD) and bovine respiratory disease (BRD) are the most common causes of morbidity and mortality in young dairy cattle [1-4]. Early forecasting of NCD and BRD in calves is considered to be a prerequisite for their successful treatment [5]. It is widely accepted that clinical examination of calves with BRD is not an appropriate and sufficient way to assess damage severity and functional changes in the lung tissue [5]. Therefore, there is a need for new biomarkers to forecast the individual risk of BRD development in calves to obtain higher accuracy in management strategies [3,6,7]. One of the BRD predictors of potential interest for newborn calves is the total protein concentration in blood serum [2]. However, limited information is available regarding the relationship between BRD and the development of protein metabolism indicators in calves during the neonatal period. The cattle neonatal period is characterized by intensive growth and adaptation of the entire organism’s systems to the intrauterine existence conditions. During this time, the organism’s requirement for protein is particularly high. Protein is the most important plastic component of metabolism, an indispensable source of biogenous nitrogen required in growth, regeneration, and immunity. Protein homeostasis formation requires the metabolic potential mobilization in a newborn [8-10] and is accompanied by an increased load on the protein synthesis systems. Metabolic adaptation disruption in calves during the neonatal period could lead to the development of various infectious and non-infectious diseases [11,12].

To assess the state of protein metabolism in calves in routine laboratory practice, the following indicators are often used: Total protein concentration in blood serum, albumin and globulin protein fractions, urea concentration, and creatinine concentration. However, these indicators do not provide a complete picture of the protein homeostasis state. Nucleoli are responsible for protein synthesis in somatic cells and ensure rRNA production and ribosomes formation [13,14]. Chromosome zones, where ribosomal gene clusters are localized, are called the nucleolus organizer regions (NORs) or nucleolus organizers. The number of active nucleolus organizers correlates with the number of mature rRNA [15] and, therefore, could be used to assess the protein synthesis system viability in cells and tissues. On the other hand, the nucleolus formation process is subject to complex regulation because it must be highly responsive to various cellular stimuli, such as nutrient availability [16-19]. An objective assessment of the protein metabolism state in newborn calves could only be provided based on its dynamic characteristics [11,12,20]. At the same time, it is necessary to different physiological processes associated with the calves’ metabolic adaptation during the neonatal period and pathological processes caused by their exposure to infectious and non-infectious factors.

This study aimed to conduct a comparative assessment of the dynamics of the main indicators of protein metabolism and lymphocyte NORs activity in healthy calves and in calves with BRD during the 1^st^ month after birth, and their relationship with the development of BRD.

## Materials and Methods

### Ethical approval

Blood samples were collected as per standard sampling procedures without any harm to the animals. Approval from the Institutional Animal Ethics Committee was not required; the study did not affect normal animal physiology.

### Animal materials and study design

The studies were performed during the winter, keeping cattle stalled period. Thirty calves of the red-motley Holstein breed, selected by random sampling, were included in the study. The animals were kept in a dispensary with 5-6 heads per cage for 10-20 days. Then, they were transferred to the calf house group cages (5-8 heads each), where they stayed until they reached 2-4 months of age. Newborn calves received colostrum from their mothers from nipple fountains 3 times a day. For the first 10 days, colostrum (then milk) was administered in the amount of 1/10 of the animal’s weight; from the 10^th^ to the 20^th^ day, 1/5 of the animal’s weight was administered; from the 21^st^ day onward, whole milk replacer or inverse was given to the animals. From 10 to 12 days old, calves were taught to eat hay, and from 18 to 20 days old, they were taught to eat concentrated feed.

The calves’ health status was assessed daily by determining body temperature, cardiac contractions, rate of respiratory movements, diarrhea (presence/absence), cough, nasal discharge, eye discharge, behavior alterations, sucking reflex, and appetite. The calves were evaluated using the WI clinical scoring system, developed by veterinarians at the University of Wisconsin in Madison [21], who conducted the thoracic cage auscultation.

### Collection of samples

For laboratory studies, venous blood samples were extracted from all calves in the morning before feeding on the 1^st^, 7^th^, 14^th^, and 28^th^ days after birth.

Blood samples from all calves were obtained using the jugular vein puncture and were collected into sterile vacuum tubes with ethylenediaminetetraacetic acid and tubes without anticoagulant. After clotting for 1 h at room temperature, blood samples without anticoagulant were centrifuged (UC-1612, ULAB, China) at 4000× g for 10 min at room temperature and sera were carefully harvested and stored at –20°C until biochemical analysis.

### Biochemical analysis

Quantitative analysis of total protein and immune globulin in serum was conducted by spectrophotometry (UV-1700, Shimadzu, Japan) according to the procedure modified from the relevant literature [22]. Serum urea and creatinine levels were determined by an automated biochemistry analyzer (Hitachi-902, Roche Diagnostics, Japan).

### Transcriptionally active chromosome NORs determination in the interphase lymphocyte nuclei

Transcriptionally active chromosome NORs in the lymphocytes interphase nuclei were identified using the W. Howell, D. Black method [23] by staining 50% with silver nitrate solution at 20°C temperature in the dark for 20 min, and at 37°C temperature for 5 min in a thermostat. The average number of nucleoli in a cell was determined as the ratio between the total number of nucleoli and the number of cells analyzed. More than 200 lymphocytes were analyzed using each preparation.

### Statistical analysis

Statistical results processing was performed using the Stadia 7.0 Professional (InCo, Russia) and MedCalc 17.5.3 (MedCalc Software, Belgium) software packages. Retrospectively, the animals were divided into two groups: Group I – calves that remained healthy during the entire observation period, WI clinical score ≤3 (n=23) and Group II – calves that developed BRD, WI clinical score ≥5 (n=7). ω^2^, χ^2^, and Kolmogorov’s criteria were used to check the indicators’ normality of distribution. A comparison of the median of the samples was performed using the Wilcoxon W Criterion (to identify differences between healthy and sick animals of the same age) and Wilcoxon’s W Criterion for the paired data (to identify age dynamics in each group). All data were expressed as means±standard deviation, and median indicators’ values were also provided. To identify BRD predictors in calves, receiver operating characteristic (ROC) analysis was used according to the DeLong *et al*. method [24]. The following ROC curve parameters were analyzed: Area under the curve, sensitivity (%), specificity (%), and cutoff point. Factor analysis was performed according to Kulaichev’s [25] recommendations. The relationship between variables was identified using the Spearman correlation coefficient. The most significant (general) factors were distinguished based on the analysis of the scree plot. The null hypothesis was rejected at p<0.05.

## Results

**Figure-1:**
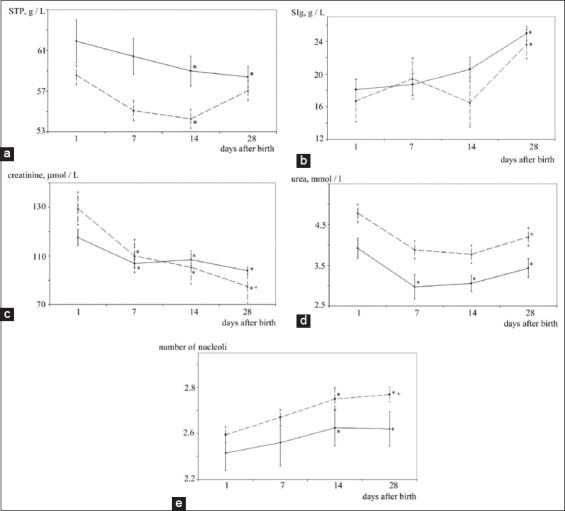
(a-e) Protein metabolism dynamics in blood serum and the number of nucleoli in lymphocytes in healthy and bovine respiratory disease developed calves in the 1^st^ month of life indicators (a – total protein, b – immunoglobulins, c – creatinine, d – urea, e – the number of nucleoli in lymphocytes). __- healthy animals, ---- - sick animals, Statistically significant differences compared to baseline values (age of 1 day): *p<0.05. Statistically significant differences between Groups I and II:^+^p<0.05.

Serum total protein (STP) concentration in calves on the 1^st^ day of life in Group II was 58.6±3.0 (60.2 median) g/l and did not significantly differ from the values in Group I (Table-1). On the 7^th^ day after birth, STP remained almost unchanged from the baseline in both groups. On the 14^th^ day after birth, STP was significantly decreased compared to those on day 1 in both Group I (by 2.8%, p<0.05) and Group II (by 8.6%, p<0.05), and amounted to 58.7±1.4 (58.5 median) g/l and 54.3±1.8 (55.0 median) g/l, respectively. On the 28^th^ day of life, STP in Group I remained below the initial level by 4.8% (p<0.05); in Group II, it increased to 57.1±0.7 (56.8 median), which did not significantly differ from that of the 1^st^ day of life. Thus, in Group I, STP decreased during the 1^st^ month of life, and in Group II, the STP dynamics were of undulating character (Figure-1a). It should be mentioned that throughout the entire study period, STP values in calves of Groups I and II remained within the reference intervals for their breed and age [20,26,27].

**Table 1:** Blood serum protein metabolism indicators and the number of transcriptionally active chromosome nucleolus organizer regions in the lymphocytes interphase nuclei in calves during the 1^st^ month of life (median values [mean±standard deviation]).

Parameter	Calves’ age			

	1 day	7 days	14 days	28 days
Total protein, g/l	60.2 (61.6±2.0) 60.2 (58.6±3.0)	59.7 (60.1±1.6) 57.3 (55.1±2.0)	58.5* (58.7±1.4) 55.0 * (54.3±1.8)	57.3* (58.4±0.9) 56.8 (57.1±0.7)
Immune globulin, g/l	18.0 (18.1±1.3) 17.2 (16.7±2.1)	19.7 (18.7±1.3) 19.1 (19.4±2.0)	20.9 (20.5±1.5) 20.9 (20.5±1.5)	25.7 * (25.0±0.8) 23.0 * (23.6±1.5)
Creatinine, μmol/l	23.0 * (23.6±1.5) 129 (149±38)	102 * (104±18( 105* (110±16)	103* (107±23) 106 * (101±18)	99 * (98±13) 86 *† (85±7)
Urea, mmol/l	4.0 (3.9±1.2) 4.1 (4.8±1.5)	2.5* (3.0±1.4) 3.2 (3.9±1.8)	2.9 * (3.1±0.9 3.0 (3.8±1.8)	3.3* (3.4±1.1) 4.2† (4.2±1.1)
Transcriptionally active NOR, units	2.4 (2.4±0.3) 2.6 (2.6±0.3)	2.5 (2.5±0.3) 2.8 (2.8±0.4)	2.6 * (2.7±0.5) 3.1 (2.9±0.4)	2.6 * (2.6±0.3) 2.8*† (2.9±0.3)

Above the line: Group I (n=23), under the line: Group II (n=7). Statistically significant differences compared to baseline values (age of 1 day):

* p<0.05. Statistically significant differences between Group I and Group II: †p<0.05. NOR=Nucleolus organizer regions

Immune globulins serum content (Serum immune globulin [SIg]) in calves in Group II on the 1^st^ day after birth was 16.7±2.12 (17.2 median) g/l and did not significantly differ from the level in Group I, i.e., 18.1±1.3 (18.0 median) g/l; these did not go beyond the range of normal values for this breed and age [26,27]. During the first 2 weeks after birth, the calves’ SIg fluctuated within the statistical error limits and did not significantly differ between the groups (Table-1). On the 28^th^ day, SIg increased significantly compared to the level on the 1^st^ day of life both in Group I (by 42.8%, p<0.05) and Group II (by 33.7%, p<0.05), and amounted to 25.0±0.8 (25.7 median) g/l and 23.6±1.5 (23.0 median) g/l, respectively.

Creatinine serum content in calves in Group II on the 1^st^ day of life was 149.0±37.8 (129.0 median) µmol/l and did not significantly differ from the level in Group I (Table-1). Creatinine concentration in the calves’ blood serum was decreasing during the 1^st^ month of life in both groups, reaching its minimum on the 28^th^ day after birth. However, throughout the entire observation period, the creatinine serum concentration values in calves of Groups I and II did not go beyond the range of reference values for a given breed and age [28,29]. On the 28^th^ day after birth, the creatinine serum content in Group II was 13.1% (p<0.05) lower than that in Group I.

Urea serum content in calves on the 1^st^ day after birth did not differ significantly between the groups and constituted 3.9±1.2 (4.0 median) mmol/l in Group I and 4.8±1.5 in Group II (4.1 median) mmol/l, which corresponded to the age norm for those animals [29]. On the 7^th^ day, urea concentration in the Group I calves’ blood serum markedly decreased by 37.5% compared to that on the 1^st^ day of life; despite the upward trend on the 14^th^ and 28^th^ days of life, the urea concentration remained below the reference values interval adopted for this breed and age until the end of the observation period [29]. Urea serum content in Group II calves did not change statistically over the entire observation period compared to the level on the 1^st^ day of life; on the 28^th^ day, it constituted 4.2±1.1 (4.2 median) mmol/l, which was 27.3% (p<0.05) higher than that in Group I (Table-1).

The number of the chromosomes in transcriptionally active NORs in the interphase lymphocyte nuclei in 1-day-old calves did not significantly differ between the groups and amounted to 2.43±0.32 (2.38 median) units in Group I and 2.59±0.33 (2.57 median) units in Group II. On the 7^th^ day after birth, the number of active NOR in calves, both in Group I and Group II, remained almost unchanged compared with the level on the 1^st^ day (Table-1). Seven-day-old calves, as well as 1-day-old calves, were characterized by the number of nucleoli that did not significantly differ between groups. A significant increase in the numbers of active NORs compared to that on the 1^st^ day of life was found in calves in both groups at the age of 14 and 28 days. However, in the Group II animals, the NOR activity on the 28^th^ day of life exceeded that of calves from Group I by 6.5% (p<0.05) (Figure-1e).

**Figure-2:**
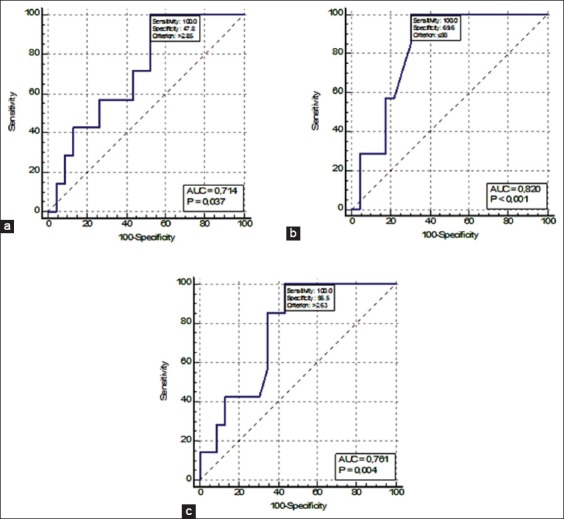
(a-c) Receiver operating characteristic curves of late pneumonia predictors (a – urea, b – creatinine, c – nucleolus organizer region activity).

According to the results of the ROC analysis, early BRD could not be identified among the studied indicators (1-14 days indicator predictors). Late BRD predictors could be the urea and creatinine concentration, as well as the NOR activity, on day 28 (Figure-2).

The sensitivity of all indicators was 100%, which makes them the perfect BRD identifiers. These tests’ specificity was relatively low (from 47.8% for urea to 69.6% for creatinine). The predictors’ quality was rated as good (urea and NOR activity) and very good (creatinine). Indicators’ critical values demonstrating the presence of a pathological process in an organism were as follows: Urea, >2.85 mmol/l; creatinine, <93.0 μmol/l; and number of active NOR, >2.63.

A significant positive correlation between the STP and the SIg content (r=0.832) was observed in the 1-day-old calves. On the 7^th^ day of observation, the relationship between STP and SIg indices was lower compared to that on the 1^st^ day (r_s_=0.513). Functional relationships between the other variables were not identified within the indicated time of observation.

When selecting the main components, the following eigenvalues of factors and explained dispersion share values were obtained (Table-2). According to the Kaiser criterion, factors with eigenvalues higher than 1 are considered as significant; their share on different observation days ranged from 44.22% to 65.02% of the explained indicators dispersion. An increase in the number of significant factors up to three raises the total share of explained variance up to 76.29-83.67%; therefore, the effect of three factors on the protein metabolism indicators was analyzed.

**Table 2:** Eigenvalues and explained dispersion factors share in calves at 1-28 days of life.

Factor	1	2	3	4	5
1^st^ day					
Eigenvalue	1.902	1.349	0.932	0.670	0.146
Dispersion, %	38.04	26.98	18.65	13.4	2.93
Accumulated dispersion, %	38.04	65.02	83.67	97.07	100.00
7^th^ day					
Eigenvalue	1.762	1.282	0.932	0.618	0.406
Dispersion, %	35.24	25.64	18.65	12.36	8.12
Accumulated dispersion, %	35.24	60.88	79.52	91.88	100.00
14^th^ day					
Eigenvalue	2.211	0.991	0.803	0.618	0.377
Dispersion, %	44.22	19.82	16.05	12.36	7.45
Accumulated dispersion, %	44.22	64.04	80.09	92.46	100.00
28^th^ day					
Eigenvalue	1.681	1.263	0.870	0.743	0.442
Dispersion, %	33.61	25.27	17.41	14.87	8.85
Accumulated dispersion, %	33.61	58.88	76.29	91.16	100.00

**Figure-3:**
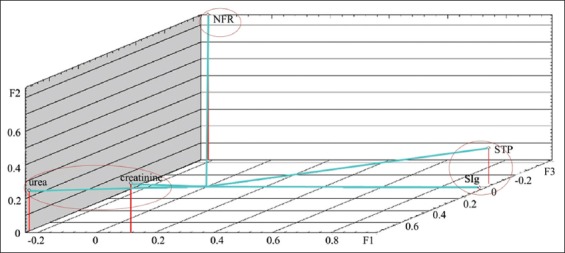
Protein metabolism indicators factor loads in calves of 1 day of age on the main components axis.

**Figure-4:**
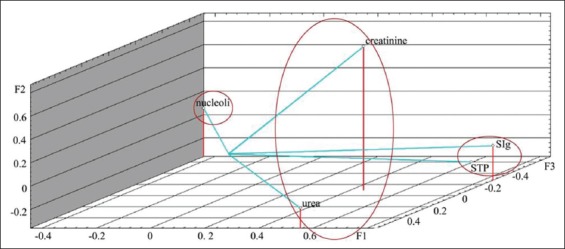
Protein metabolism indicators factor loads in calves of 7 days of age on the main components axis.

**Figure-5:**
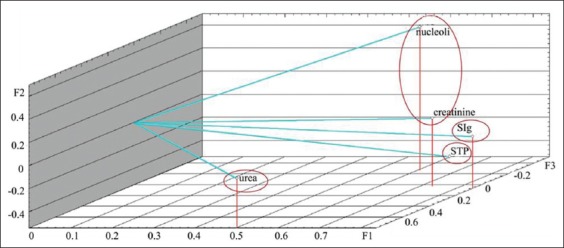
Protein metabolism indicators factor loads in calves of 14 days of age on the main components axis.

**Figure-6:**
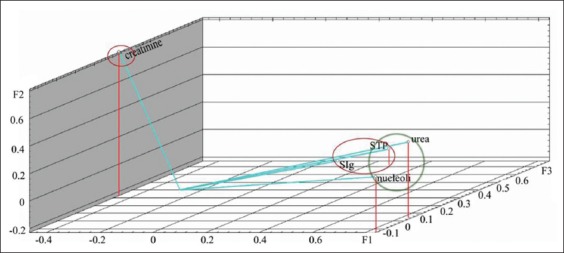
Protein metabolism indicators factor loads in calves of 28 days of age on the main components axis.

The calculation of factor loads on the analyzed variables is presented in Table-3. Graphs demonstrating factor loads on indicators on days 1 and 7 are presented in Figures-3 and 4. As shown, characters under study within the main components space create three clearly distinguishable groups: STP+SIg, urea+creatinine, and nucleoli. The analyzed properties are projected primarily on one of the three main factors, which facilitates the task of interpreting factors in terms of initial variables. On the 14^th^ day of observation, functional relationship between the protein metabolism indicators was not identified. The analyzed characters were redistributed within the main components space, i.e., STP and SIg; urea and creatinine did not constitute unitary groups (Figure-5). On the 28^th^ day of observation, correlation was found between the NOR activity and the urea level (r_s_=0.539); no functional relationship was identified between the other protein metabolism indicators. The graph of indicators factor loads is presented in Figure-6. As shown in Figure 6, STP and SIg indicators again formed a unitary group within the factor axes space; the new group combined the nucleoli and the urea indicators.

**Table 3:** Factor loads on the protein metabolism indicators in calves in 1-28 days of life.

Indicators	STP	SIg	Transcriptionally active NOR	Urea	Creatinine
1^st^ day					
Factor 1	0.933	0.956	0	0	0
Factor 2	0	0	0	0.741	0.768
Factor 3	0	0	0.863	0	0
7^th^ day					
Factor 1	0.597	0.836	0	0.554	0
Factor 2	−0.599	0	−0.705	0.582	0
Factor 3	0	0	0.543	0.558	0.874
14^th^ day					
Factor 1	0.598	0.838	0.605	0.504	0.728
Factor 2	−0.519	0	0	0.803	0
Factor 3	−0.539	0	0.699	0	0
28^th^ day					
Factor 1	0	0	0.840	0.827	0
Factor 2	−0.757	−0.776	0	0	0
Factor 3	0	0	0	0	0.817

NOR=Nucleolus organizer regions, STP=Serum total protein, SIg=Serum immune globulin

**Figure-7:**
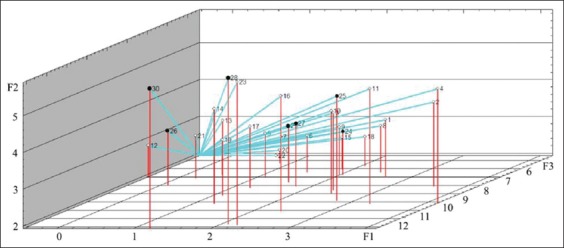
Study objects (calves of 1 day of age) within the main components space. ° - healthy animals (No. 1-23), • - diseased animals (No. 24-30).

Figures-7,10 show the distribution of the study objects (calves) within the main components space. Species that subsequently developed BRD (No. 24-30, highlighted in color) do not constitute a separate group which indicates that features in the protein metabolism status during days 1-14 of life were missing in the remaining healthy and diseased calves. On the 28^th^ day of life, compared with the preceding observation period, diseased calves created a more compact group within the main components space, but it is not separated from the total sample (Figure-10).

**Figure-8:**
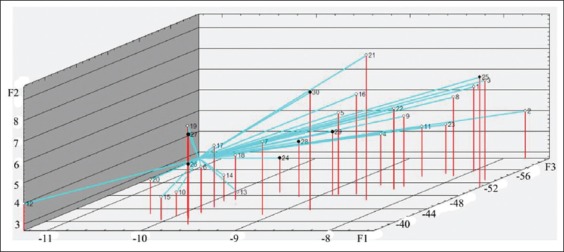
Study objects (calves of 7 days of age) within the main components space. ° - healthy animals (No. 1-23), • - diseased animals (No. 24-30).

**Figure-9:**
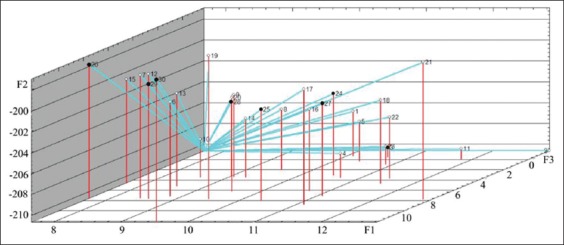
Study objects (calves of 14 days of age) within the main components space. ° - healthy animals (No. 1-23), • - diseased animals (No. 24-30).

**Figure-10:**
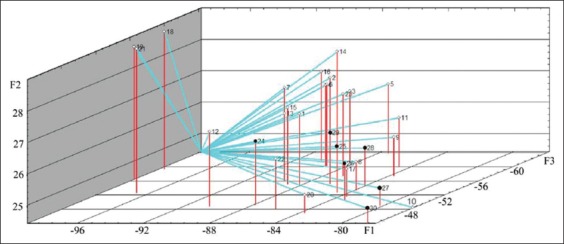
Study objects (calves of 28 days of age) within the main components space. ° - healthy animals (No. 1-23), • - diseased animals (No. 24-30).

Analysis of the factor loads distribution on the indicators supports the following interpretation of the three operative factors. Factor 1 exerts the maximum load on the STP and SIg indicators during the first 14 days of observation and on the nucleolus and urea indicators from the 14^th^ to the 28^th^ days. Thus, it could be interpreted as preservation of the protein metabolism indicators at the level specified on the 1^st^ day after birth. Factor 2 mainly affects the STP and urea indicators and could be interpreted as the protein catabolism intensity. Factor 3 mainly influences the active NOR level and is probably associated with *de novo* protein synthesis activation.

## Discussion

Blood plasma is a dynamic equilibrium system, which generally reflects the state of organism like a mirror. Total protein is an integral indicator of protein metabolism. Hypoproteinemia in ruminants is observed under malnutrition, infectious, inflammatory, and oncological diseases, and cicatricial digestion, liver, kidney, and intestinal function disorders. Hyperproteinemia could be caused by excessive fluid loss, liver pathologies, and acute and chronic infectious diseases; it develops due to the increased production of immunoglobulins [30].

Our study on healthy animals demonstrated a decrease in the blood serum protein content starting from the 14^th^ day, which continued until the end of the month. A similar effect in the 1^st^ month of life of calves was reported by several authors [10,25,31]. In the diseased calves, STP minimum concentration was identified on the 14^th^ day after birth followed by recovery to the level recorded on the 1^st^ day. STP concentration average values were lower in diseased calves than in healthy calves; however, no statistically significant differences were found, which is the same as the findings of Villarroel *et al*. [26].

A special position among proteins is occupied by immunoglobulins (antibodies). In ruminants, the desmochororial placenta does not allow the mother’s immunoglobulins to enter the embryo blood. The blood of a newborn calf is practically free of antibodies until the first feed with colostrum; colostrum is the only source of immunoglobulins [10,32]. Information about the level of antibodies in the blood of newborn calves is contradictory. Tóthová *et al*. [10] and Villarroel *et al*. [26] noted a decrease in the Ig concentration within the 1^st^ month after birth; Piccione *et al*. [31] and Hulbert and Moisá [33] observed a decrease in the SIg content from the 1^st^ to the 15^th^ days followed by a gradual increase by the 20^th^ day of life.

We found positive dynamics of changes in the blood immunoglobulin content of both healthy and BRD developed calves. Healthy animals demonstrated a tendency for an increase in the antibody concentration throughout the entire observation period; the statistically significant increase in the studied parameter was observed on day 28.

Calves with bronchopulmonary pathology manifested the colostral antibody reserves depletion by the end of the 2^nd^ week (Figure-1b). However, immunoglobulin concentration was restored to the level of the control group on the 28^th^ day, probably due to activation of antibody synthesis by plasma blood cells.

Thus, a decrease in STP concentration is associated with degradation of colostral antibodies [10,31,34] and serum albumin, which has a half-life of 14-16 days [10]. An increase in STP with the diseased calves on the 28^th^ day (compared to day 14) can be explained by the production of antibodies and the rise in the serum protein γ-globulin fraction.

Creatinine is synthesized as a result of endogen metabolism in muscles. Creatinine is excreted with urine; its concentration in serum does not depend upon nutrition. Creatinine concentration increases only at serious damage [35]. In calves, a significantly high creatinine serum concentration was observed after birth (256±106 μmol/L); the value normalized by the 4^th^ day of age (108±28 μmol/L) [36]. Similar development was identified by Maach *et al*. [37], and after the 15^th^ day of age, an increase in creatinine concentration until the age of 60 days was observed, which raised the value to 146.7±23.9 μmol/L. The research by Mohri *et al*. [34] demonstrated that concentration of creatinine in Holstein calves decreased from the 1^st^ to the 70^th^ days of age.

The creatinine concentration decrease from birth to the end of the 1^st^ month of life indicates how the newborns adapt to changes and maintain homeostasis [38,39]. Results obtained in our study do not contradict the data of other authors; creatinine concentration in the blood of healthy and diseased calves decreased throughout the entire observation period (Table-1, Figure-1c). At BRD, the increase in muscle mass slows down, and physical activity decreases, resulting in a decrease in the creatinine blood serum concentration in the diseased calves compared to the same indicator in the healthy calves on the 28^th^ day of the experiment (Table-1, Figure-1c).

Urea concentration in blood depends on nutrition; diagnostically, it is also important when exposed to kidney diseases [35,36]. An increased concentration in urea in the calves’ serum indicates the proteins increased catabolism and results in long-lasting diarrhea [36]. Urea concentration in calves slightly decreased from birth to the age of 60 days, when it reached 2.7 mmol/L [40]. We also found a decrease in urea blood levels in healthy calves during the 1^st^ month of life (Table-1, Figure-1d). The level of urea did not change in the organisms of the diseased animals for 28 days, even with a decrease in protein content (Table-1, Figure-1d), apparently due to the organism general intoxication against the BRD background.

The number of NORs, like the chromosome number, is a characteristic feature for the species. However, within the species nucleoli, number, size, and shape vary depending on an organ, cell type, and cell physiological state, as well as on morphological features of nucleoli reflecting the intensity of activity thereof [41,42]. Under stress conditions, it affects cell-cycle progression and/or intracellular energy status, such as nutrient deprivation; ribosome subunit biosynthesis alteration is a strategy that could preserve cellular energy homeostasis [43]. NORs in *Bovidae* are present in five autosome pairs (2, 3, 4, 11, and 25) in the distal parts of the q arms [44]. The number of nucleoli in a cell nucleus is determined by the number of active NOR. It may be equal to the number of those NOR chromosomes, but normally, it is lower [45].

We discovered that the calves’ peripheral blood lymphocytes NORs activity was not going out beyond the norms during the entire observation period. The increase in the number of nucleoli by day 14 (Table-1, Figure-1e) indicates activation of the B-lymphocyte protein-synthesizing system. Thus, results of the cytological analysis 2 weeks before the registered increase in the blood serum immunoglobulins concentration in calves could determine the lymphocyte readiness to synthesize antibodies. On the 28^th^ day of life, calves with the developed BRD had a significantly higher number of nucleoli in lymphocytes than that in healthy animals, which indicates some additional stimulation of the B-lymphocytes protein-synthesizing function under the pathological bronchopulmonary conditions.

Protein metabolism in the newborn calves’ organisms is regulated by three types of factors: Maintaining the constant protein concentration in plasma, its decomposition, and *de novo* synthesis. Separation of connections and rearrangement of analyzed parameters within the main components space at the end of the second until the beginning of the 3^rd^ week of the experiment indicates that the organism turn to production of its own proteins induced by depletion of the protein reserves synthesized during the prenatal period or obtained through colostrum.

Diseased animal groupings in the factor axes space into a fairly compact cluster observed on day 28 of the experiment (Figure-10) indicate characteristic changes in the protein metabolism observed at the BRD development (decrease in STP, SIg and creatinine, and increase in urea concentration). Diseased animals’ separation according to protein metabolism indicators in a separate group from the remaining healthy animals was not performed.

## Conclusion

The STP and SIg concentration decrease in the calves’ blood plasma during the 1^st^ month appears to be an option of the physiological norm and is associated with reduction of serum albumin content and of colostral antibodies in blood plasma. The beginning of the 3^rd^ week is a critical period in the calves’ life, because during this period there is a turn of metabolism from using proteins primarily synthesized *in utero* or obtained from colostrum to synthesizing new proteins under the changed (extrauterine) environmental conditions. Maturity of the organism protein-synthesizing systems determines the animal’s state of health during this period.

Significant differences in the protein metabolism values and dynamics of indicators between healthy calves and calves with developed BRD were not identified. Alterations in the characteristics studied are the result, but not the cause of BRD. Thus, they could not act as predictors of the cattle respiratory diseases, as was previously pointed out by Windeyer *et al*. [2].

An increase in the number of active NORs under conditions of the BRD comprehensive clinical picture could be a favorable forecasting indicator, since it demonstrates maturity and sufficient adaptive potential of the humoral immunity element and of the organism’s immune system as a whole. Antibodies production function by B-lymphocytes shows sufficient autonomy from the protein metabolism general state, since the antibody synthesis and an increase in their concentration in blood plasma occur against the background of the STP concentration decrease. Consequently, protection against foreign protein and genetic material, i.e., implementation of the immune system functions, is a more important task for the organism than ensuring growth processes during the neonatal period.

## Authors’ Contributions

VK and AC conceived and designed the study. KE and AC collected samples and performed the experiments. KE, AC, and VS performed the laboratory analyses. EK and VK performed the statistical analyses. EK drafted the manuscript. AC and VS revised the manuscript critically. All authors read and approved the final manuscript.
